# A Novel Pathway of TEF Regulation Mediated by MicroRNA-125b Contributes to the Control of Actin Distribution and Cell Shape in Fibroblasts

**DOI:** 10.1371/journal.pone.0017169

**Published:** 2011-02-11

**Authors:** Olga Gutierrez, Maria T. Berciano, Miguel Lafarga, Jose L. Fernandez-Luna

**Affiliations:** 1 Unidad de Genetica Molecular, Hospital Universitario Marques de Valdecilla, Instituto de Formacion e Investigacion Marques de Valdecilla, Servicio Cantabro de Salud, Santander, Spain; 2 Departamento de Anatomia y Biologia Celular, Universidad de Cantabria, Santander, Spain; French National Center for Scientific Research - Institut de Biologie Moléculaire et Cellulaire, France

## Abstract

**Background:**

Thyrotroph embryonic factor (TEF), a member of the PAR bZIP family of transcriptional regulators, has been involved in neurotransmitter homeostasis, amino acid metabolism, and regulation of apoptotic proteins. In spite of its relevance, nothing is known about the regulation of TEF.

**Principal Findings:**

p53-dependent genotoxic agents have been shown to be much more harmful for PAR bZIP-deficient mice as compared to wild type animals. Here we demonstrate that TEF expression is controlled by p53 through upregulation of microRNA-125b, as determined by both regulating the activity of p53 and transfecting cells with microRNA-125b precursors. We also describe a novel role for TEF in controlling actin distribution and cell shape in mouse fibroblasts. Lack of TEF is accompanied by dramatic increase of cell area and decrease of elongation (bipolarity) and dispersion (multipolarity). Staining of actin cytoskeleton also showed that TEF (−/−) cells are characterized by appearance of circumferential actin bundles and disappearance of straight fibers. Interestingly, transfection of TEF (−/−) fibroblasts with TEF induced a wild type-like phenotype. Consistent with our previous findings, transfection of wild type fibroblasts with miR-125b promoted a TEF (−/−)-like phenotype, and a similar but weaker effect was observed following exogenous expression of p53.

**Conclusions/Significance:**

These findings provide the first evidence of TEF regulation, through a miR-125b-mediated pathway, and describes a novel role of TEF in the maintenance of cell shape in fibroblasts.

## Introduction

Mammalian homologs of the C. elegans protein CES-2 include thyrotroph embryonic factor (TEF), albumin D-site-binding protein (DBP), and hepatic leukemia factor (HLF) [1], [2], [3] which are members of the proline- and acid-rich (PAR) subfamily of basic region leucine-zipper (bZIP) transcription factors. Mice deficient for all three PAR bZIP proteins are highly susceptible to generalized spontaneous and audiogenic epilepsies that frequently are lethal [4]. These proteins have recently been described to control the expression of many enzymes and regulators involved in detoxification and drug metabolism [5]. Additionally, we have shown that TEF regulates the expression of two genes, bcl-gS and bik, involved in the execution of apoptosis [6], [7]. Genotoxic agents such as cyclophosphamide and mitoxantrone, which induce p53-dependent apoptosis, have been shown to be much more harmful for PAR bZIP-deficient mice as compared to wild type animals [5]. However, no relationship between p53 and PAR bZIP transcription factors has been established to date.

An interesting but not well known activity of p53 is its capacity to modify cell shape. Expression of some p53 mutants and to a lesser extent wild type p53, lead to increase of average cell area, decrease of dispersion and elongation indices, and re-appearance of actin bundles in both Ras-transformed fibroblasts and epitheliocytes [8]. The key factors that are responsible for the shape of a cell are primarily the cytoskeletal structures, including microfilaments, microtubules and intermediate filaments [9], [10]. A set of intracellular molecules are likely to act in a spatially determining way to organize these shape-determining factors on a particular path. However, we know little about these signalling networks, although recent data have identified a large number of genes that influence cytoskeletal organization and morphology in Drosophila cell lines [11], [12], which indicates the complexity of the problem. Although stress fibers (bundles of actin filaments) play a central role in adhesion, motility, and morphogenesis of eukaryotic cells, the mechanism of how these and other contractile actomyosin structures are controlled and redistributed in the cell is not well understood. Additionally, several groups have established the relationship between p53 and microRNAs. These studies highlight the microRNAs miR-34a and miR-34b/c as direct, conserved p53 target genes that presumably mediate many of the activities of p53 in response to stress stimuli [13]. Responding to stresses, cells either choose to restore or reprogram their gene expression patterns, which is partly mediated by microRNA functions. In turn, these changes determine the specificity, timing, and concentration of gene products expressed upon stresses [14]. Recent evidence indicate that p53 is able to induce the expression of a number of microRNAs by either direct transcriptional activation or regulation of the microRNA processing complex. Direct targets of p53 include the already mentioned miR-34 and also miR-192, miR-194 and miR215, which then modulate MDM2 expression [15]. The proposed microRNAs that are regulated through the processing machinary include let-7, miR-200c, miR-143, miR-107, miR-16, miR-145, miR-134, miR-449a, miR-503, and miR-21 [16]. In the present study, we demonstrate that TEF is downregulated by miR-125b through activation of p53, and that this novel regulation pathway contributes to determine the actin distribution and the shape of fibroblasts.

## Results

### p53 regulates the expression of TEF

**Figure 1:**
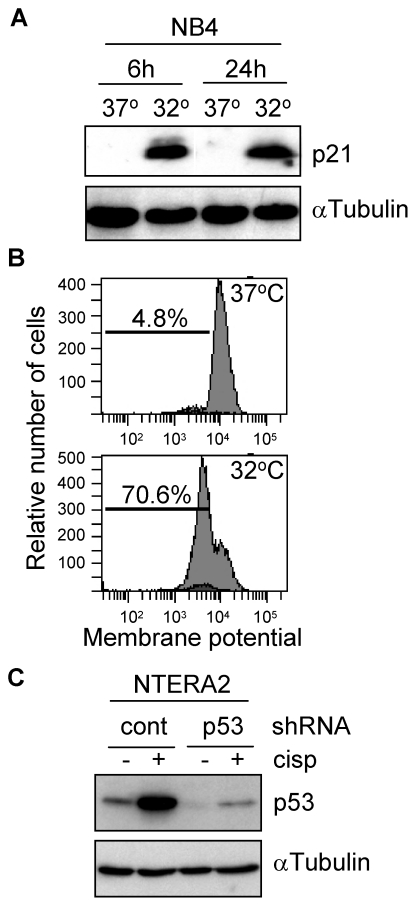
Cell models genetically modified to study p53-dependent signalling pathways. (A) NB4 cells transfected with a temperature-sensitive mutant form of p53 were incubated at the indicated conditions and the activity of p53 was determined by analyzing the protein levels of p21. (B) The proportion of apoptotic NB4 cells in response to p53 activation was determined by the loss of mitochondrial membrane potential in a flow cytometer. (C) NTERA2 cells were stably transfected with a p53-specific or a control shRNA. The knockdown efficiency was determined by western blot following incubation of cells in the presence of 12 µM cisplatin for 24 h. The expression of α-Tubulin was determined to assure equal loading.

**Figure 2:**
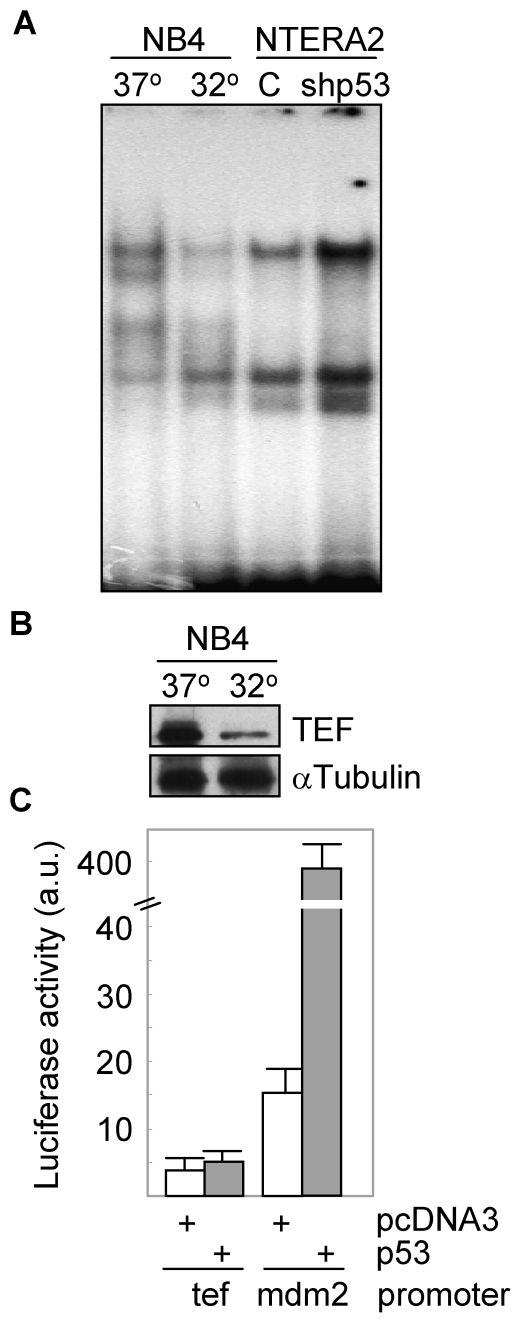
Activation of p53 downmodulates TEF. (A) Nuclear extracts from NB4 cells cultured at the indicated temperatures and NTERA2 cells transfected with control or p53 shRNAs, were obtained and analyzed for the formation of protein-DNA binding complexes by EMSA. (B) The expression of TEF was analyzed in protein lysates from NB4 cells following activation of p53 by western blot. The levels of α-Tubulin were determined to assure equal loading. (C) HEK293T cells were co-transfected with a TEF promoter-luciferase reporter vector and with p53 cDNA or empty vector (pcDNA3). The mdm2 promoter was included as a positive control of response to p53. After 24 h of transfection, cell extracts were prepared and analyzed for the relative luciferase activity. Results were normalized for transfection efficiency with values obtained with pRSV-β-gal. All data points represent the means ± SD of three independent experiments.

Genotoxic agents that induce p53-mediated apoptosis, are more harmful for PAR bZIP-deficient mice as compared to wild type animals [5]. TEF and other PAR bZIP proteins stimulate the transcription of genes encoding enzymes and regulators important for the defense against xenobiotics, thus, contributing to cell survival. We and others have described that TEF seems to be the major PAR bZIP member responsible for the expression of different target genes [4], [7]. Thus, we wanted to study whether there was a relationship between both transcription factors, p53 and TEF. We first explored whether p53 was able to control the expression of TEF. For this purpose, we used NB4 cells expressing a temperature-sensitive p53 mutant, and NTERA2 cells transfected with a p53-specific shRNA. Both cell systems were demonstrated to behave as expected. NB4 cell line upregulated p53 when shifted to 32°C as determined by the increase of p21 protein levels (Figure 1A), which correlated with an increase of more than 60% of apoptotic cells assessed by loss of mitochondrial membrane potential (Figure 1B). On the other hand, NTERA2 cells showed a downregulated expression of p53 when transfected with the specific shRNA, which was even more evident when this cell line was treated with cisplatin (Figure 1C). As expected, downregulation of p53 dramatically reduced the apoptotic response to the chemotherapeutic drug (data not shown). Both cell systems were used to study the contribution of p53 to the transcriptional activity of TEF. Interestingly, the DNA binding capacity of TEF was reduced in NB4 at 32°C and increased in p53 knockdown NTERA2 cells (Figure 2A). Moreover, shift to 32°C promoted a clear downregulation of the TEF protein levels (Figure 2B). In view of these data, we tested whether p53 was able to modulate the transcription of TEF. HEK293T cells were transfected with a construct containing the luciferase gene driven by the TEF promoter. As shown in Figure 2C, overexpression of p53 did not modify the activity of this promoter although it efficiently induced the mdm2 promoter, a known target of p53.

### miR-125b mediates TEF silencing

**Figure 3:**
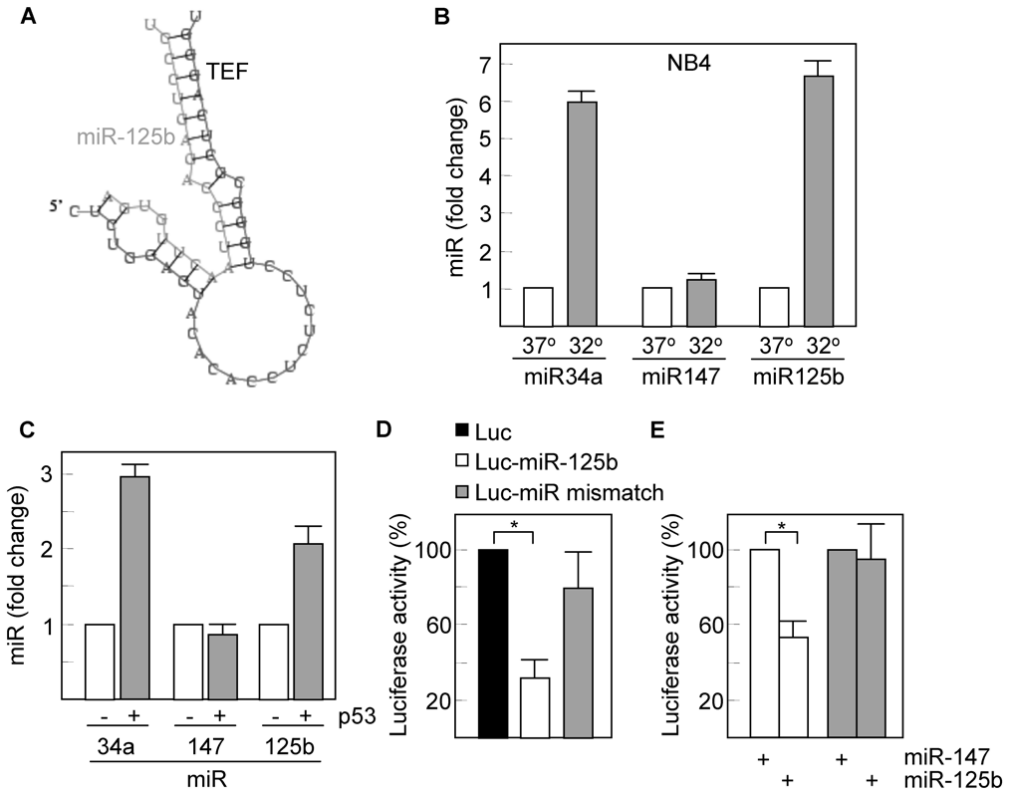
miR-125b is induced in response to p53 activation. (A) The interaction between miR-125b and the 3′UTR of TEF is shown as predicted by PICTAR. (B) Total RNA was extracted from NB4 cells cultured at 37°C or 32°C and analyzed for the expression of miR-125b by quantitative RT-PCR. A known target of p53, miR34a, and the p53-unrelated miR-147, were also included as controls. Nuclear RNA RNU6B was used as a normalization control. (C) NB4 cells were transfected with a p53 cDNA-containing vector and after 24 h, the expression of miR-125b was analyzed as above. (D) NB4 cells were transfected with a luciferase reporter vector containing the miR-125b binding sequence downstream of the luciferase gene. The same vector containing a mismatch sequence was also used as a negative control. After 16 h of transfection, cells were incubated at 32°C for another 16 h period and then extracts were prepared and analyzed for the relative luciferase activity. Results were normalized for transfection efficiency with values obtained with pRSV-β-gal. (E) HEK293T cells were co-transfected with the same luciferase reporters used in (D) and with miR-125b or control miR-147. After 24 h of transfection, cell extracts were analyzed for the relative luciferase activity. Asterisks represent significant differences (p<0.01) with the corresponding controls. Histograms show the means ± SD of three independent experiments.

Increasing evidence has suggested that miRs are translational repressor components of multiple signalling pathways. It is now known that p53 induces the expression of miRs such as the miR-34 family [17]. Therefore, we searched for miR target sequences in the TEF gene using the TargetScan and PicTar prediction programs [18], [19], and identified a highly conserved seed sequence for miR-125a and miR-125b homologs within the 3′-untranslated region of TEF that exhibit an almost perfect complementarity with both miRs (the interaction between miR-125b and human TEF is shown in Figure 3A). The maximum free energies predicted for the configuration between the target element in TEF and the seed sequences in miR-125 are –25.7 kcal/mol (miR-125a) and –27.4 kcal/mol (miR-125b) as determined by mFold analysis and consistent with authentic miR targeting [20].

**Figure 4:**
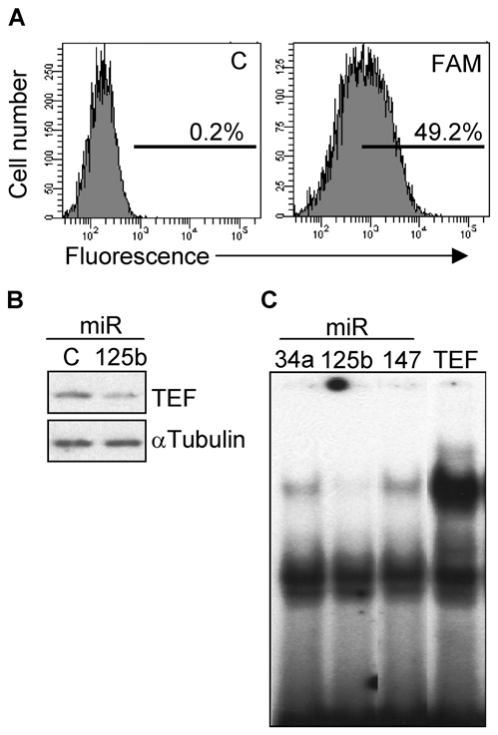
miR-125b downregulates TEF. (A) Wild type mouse fibroblasts were transfected with a FAM-labeled, random sequence control miR and the transfection efficiency was determined as the percentage of fluorescent cells by flow cytometry. C, unlabeled negative control miR. (B) Following 4 days of transfection with miR-125b or control miR, the protein levels of TEF were determined by western blot. The expression of α-Tubulin was determined to assure equal loading. (C) Nuclear extracts from wild type fibroblasts transfected with miR-34a, miR-125b or miR-147, were analyzed for the formation of TEF-DNA binding complexes by EMSA. Cells overexpressing TEF were also included to verify the specificity of the complex.

By using quantitative PCR, we then showed that activation of p53 in NB4 cells increased the expression of miR-125b to nearly the same extend as did the p53 target miR-34a, but had no effect on the levels of miR-147, used here as a p53-independent miR (Figure 3B). The role of p53 in promoting the expression of miR-125b was further confirmed by transfection of NB4 cells with a p53-containing vector. Both miR-34a and miR-125b were upregulated, whereas the levels of miR-147 remained unmodified (Figure 3C). In order to verify whether the binding site for miR-125b identified in the TEF gene can repress translation, we introduced this conserved sequence downstream of the luciferase gene in a reporter construct. Transfection of NB4 cells that express the temperature-sensitive p53 mutant with this construct and subsequent p53 activation led to a significant and specific reduction (about 3-fold) of the luciferase activity (Figure 3D). A more direct evidence was obtained following contransfection of HEK293T cells with the same luciferase construct and with miR-125b. Transfection with the specific microRNA significantly reduced the levels of luciferase activity. This reduction was not observed when cells were transfected with miR-147 or when the reporter vector contained a mismatch sequence (Figure 3E). To further extend these results we studied whether miR-125b was able to repress the expression of TEF by transfecting wild type fibroblasts with miR-125b. The transfection efficiency was in all experiments around 50%, as determined by flow cytometry using a FAM-conjugated control miR (Figure 4A). We found that miR-125b downregulated (2- to 3-fold) the protein levels of TEF (Figure 4B) which correlates with the lack of interaction between TEF and its DNA target sequence in cells overexpressing miR-125b as determined by EMSA (Figure 4C). Similar but less pronounced effects were observed with miR-125a (data not shown).

### mir-125b-TEF regulation pathway controls fibroblast cell shape

**Figure 5:**
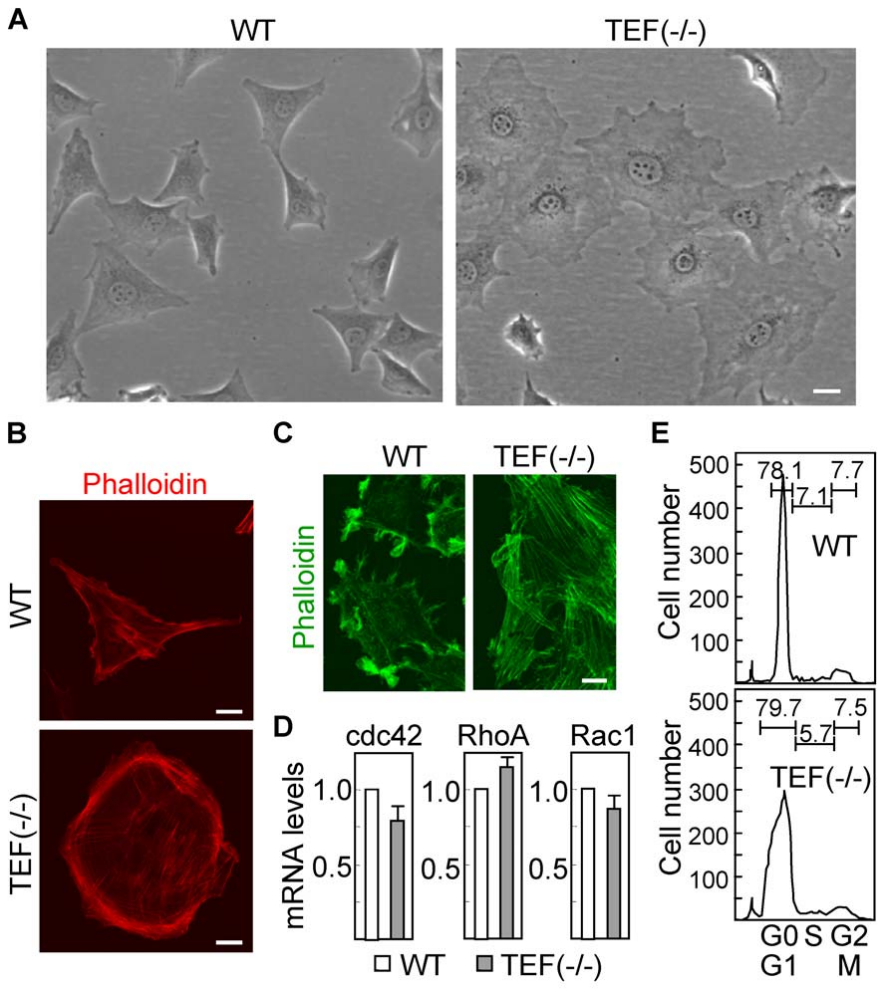
Morphological differences between wild type and TEF (−/−) fibroblasts. (A) The morphology of wild type (WT) and TEF (−/−) fibroblasts in culture was assessed by phase contrast microscopy. Scale bar: 40 µm. (B) Both cell populations were labeled red with phalloidin for staining the actin filaments and visualized by confocal microscopy. Scale bars: 20 µm. (C) Confluent cultures were mechanically disrupted, leaving an area devoid of cells, and fibroblasts were labeled green to determine the actin distribution at the leading edge. Scale bar: 20 µm. (D) The mRNA expression of different Rho GTPases was analyzed by quantitative RT-PCR. The expression levels in TEF (−/−) cells were referred to those in wild type cells. Histograms represent the mean ± SD of three independent experiments. (E) Distribution of cell cycle phases in wild type and TEF (−/−) fibroblasts was determined by flow cytometry after staining nuclei with propidium iodide. The percentage of cells in the different phases, G0/G1, S, and G2/M, is indicated.

Wild type and TEF (−/−) fibroblasts exhibit very different morphologies. Wild type cells display an elongated and stellate phenotype, whereas knockout cells appear to be larger and rounded (Figure 5A). The shape changes are generally accepted to be driven by the actin cytoskeleton, which together with accessory proteins make up the cell cortex. Consistently, we found that F-actin filaments were distributed along the periphery of knockout cells forming circumferential bundles (Figure 5B). When cells are attached to a planar substratum and moving in low density fluid cultures, there is little in the way of external forces to resist cell shape changes. We analyzed F-actin distribution in confluent cultures with a mechanically denuded area and found the staining to be localized to protrusions at the leading edge in cultures of wild type fibroblasts, and evenly distributed in filaments that were oriented more parallel to the cell edge in TEF(−/−) fibroblasts (Figure 5C). The dynamic organization of the actin cytoskeleton is regulated by small GTPases of the Rho family, in particular Rac1, RhoA and Cdc42 [21]. Since TEF is a transcription factor, we studied whether the expression of these genes was modified by the presence of TEF. As shown in Figure 5D, the mRNA levels of Rac1, RhoA and Cdc42 were very similar in both wild type and TEF (−/−) fibroblasts. Actin cytoskeleton is reorganized during mitosis to form rounded cells, and re-established after cell division allowing cells to regain a more extended shape. Therefore, we analyzed the cell cycle phase distribution in wild type and TEF (−/−) fibroblasts (Figure 5E) and found a similar pattern in both cell populations, suggesting that the difference in cell shape was not due to differences in cell division rates.

**Figure 6:**
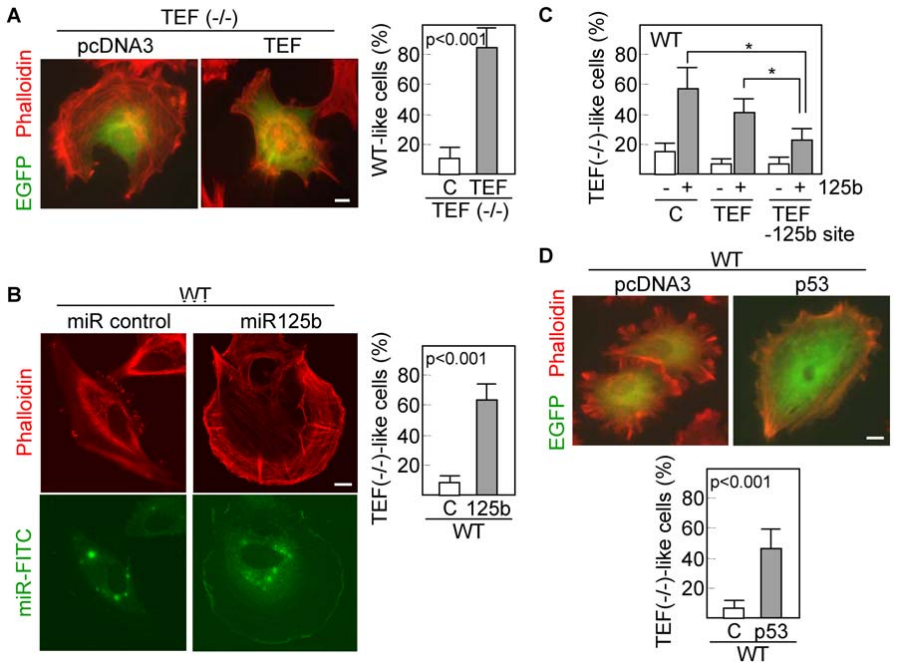
Overexpression of TEF, miR-125b or p53 modifies the cell phenotype. (A) TEF (−/−) fibroblasts were cotransfected with empty vector (pcDNA3) or TEF and an EGFP-containing vector. After 48 h of transfection, cells were labeled red with phalloidin and visualized by confocal microscopy. (B) Wild type cells were cotransfected with control miR or miR-125b and a FAM-labeled miR. Actin filaments were stained with phalloidin 48 h after transfection. Green and red fluoresce images were collected from different focal planes. (C) Wild type fibroblasts were cotransfected with a TEF cDNA lacking the miR-125b binding sequence (TEF –125b site) and miR-125b and then, the number of cells having a TEF(−/−)-like morphology was quantitated. Unmodified TEF was also included for comparison. Asterisks represent significant differences (p<0.01) with the corresponding control (empty vector transfected cells). (D) Wild type fibroblasts were cotransfected with pcDNA3 or p53 and an EGFP-containing vector and analyzed as described above. Scale bars: 10 µm. Quantifications were performed in triplicate, counting a minimum of 100 total cells each. C, control transfected cells. Histograms represent the mean ± SD.

To further confirm that TEF is a relevant factor to control the distribution of actin filaments in fibroblasts, we transiently cotransfected TEF (−/−) cells with TEF and EGFP. The transfection efficiency was about 20% as determined by flow-cytometric quantification of EGFP-positive cells. Of these, almost all (more than 80%) restored the actin filament pattern characteristic of wild type cells (Figure 6A). Moreover, when wild type fibroblasts were transfected with miR-125b precursor, actin filaments were redistributed along the cell periphery and fibroblasts acquired a rounded shape in more than 60% of transfected cells (Figure 6B) resembling the morphology of TEF (−/−) fibroblasts. To establish that the effect of miR-125b on the distribution of actin filaments was mediated through downregulation of TEF, we flow sorted DsRed-positive wild type fibroblasts cotransfected with a TEF cDNA lacking the mir-125b binding site, which were subsequently transfected with miR-125b. Consistently, overexpression of TEF significantly (p<0.01) reduced the number of TEF(−/−)-like cells as compared with fibroblasts transfected with either an empty vector or an unmodified TEF cDNA (Figure 6C). In addition, a similar but less pronounced morphological pattern was observed in about 50% of cells transfected with p53 and EGFP (Figure 6D). These data suggest that the p53-miR-125b-TEF axis has a significant influence on cytoskeletal organization and cell shape.

## Discussion

In vivo models have demonstrated that mice deficient in TEF, DBP and HLF, are hypersensitive to genotoxic drugs such as cyclophosphamide and mitoxantrone, which induce p53-dependent apoptosis [5]. We have shown that p53 downmodulates the expression of TEF not by a direct transcriptional mechanism but through a microRNA, miR-125b. The p53-mediated regulation of miR-125b is consistent with recent data showing that miR-125 served as a tumor suppressor microRNA in tumor cells [22] and that miR-125 is upregulated along with the p53 targets miR-34a-c, in K562 cells in response to cisplatin [23], a p53-dependent apoptotic inducer [24]. In addition, it has been shown that miR-125b is a negative regulator of p53 [25], which would suggest a negative feedback loop involved in the control of this tumor suppressor. However, the same authors described that miR-125b is downregulated in zebrafish embryos following p53 activation by DNA-damaging agents. Furthermore, in other systems such as leukemia cells, p53 protein levels were not altered by miR-125b [26]. All these data reveal the complexity of this regulatory network which may be influenced by the cellular context and the activating signals received by the cells. The relevance of microRNAs in mediating the activities of p53 is gaining support by a number of recent publications. One of the most elegant works provided evidence that miR-192, miR-194 and miR-215 can be transcriptionally activated by p53 [15]. These microRNAs in turn downmodulate MDM2, a negative regulator of p53, enhancing the activity of this tumor suppressor gene, which may be a relevant mechanism to control the genotoxic effects of stress stimuli in different cell systems.

We have also demonstrated that TEF regulates the distribution of actin filaments and the shape of fibroblasts. In line with the TEF-mediated pathway described here, both p53 and miRs have been involved in cell shape and cytoskeleton remodeling [8], [27], [28]. Recent data show that JMY, a transcriptional co-factor that regulates the p53 response, represents a new class of multifunctional actin assembly factor [29], and that LIMK2, a p53-target gene upregulated by DNA damage, regulates actin dynamics [30]. Taken together, all these data suggest that a number of factors, acting downstream of p53, ensure proper execution of checkpoint arrest by modulating the dynamics of actin polymerization. Interestingly, we observed that the absence of TEF in mouse fibroblasts was accompanied by increase of cell area, decrease of elongation (bipolarity) and dispersion (multipolarity), and appearance of circumferential actin bundles. Cellular activities, including cell shape, internalization of membrane vesicles, migration and cell division depend on the control of actin dynamics by numerous proteins, mainly Rho GTPases [31]. We showed no significant differences in the mRNA levels of RhoA, Rac1 and Cdc42 between wild type and TEF (−/−) fibroblasts. However, although most studies have concentrated on these three Rho proteins, the family contains more than twenty members, some of which seem to have unique roles in the regulation of actin dynamics [32]. Therefore, further studies need to be done to elucidate if any Rho GTPase is a target of TEF or alternatively whether key proteins of signal transduction cascades from membrane receptors to Rho proteins or from Rho proteins to microtubules are transcriptionally regulated by TEF.

Consistent with our previous findings, genetic manipulation of the p53-miR-125b-TEF axis directed the cells toward the expected morphological profile characteristic of either wild type or TEF (−/−), phenotypes. To this end, expression of some p53 mutants and to a lesser extent wild type p53, lead to increase of average cell area, decrease of dispersion and elongation indices, and re-appearance of actin bundles [8]. p53 expression is elevated in damaged neurons in acute models of injury such as ischemia and epilepsy and in brain tissue samples derived from patients with chronic neurodegenerative diseases, which is consistent with data showing that the absence of p53 protects neurons from a wide variety of acute toxic insults [33]. This is in good agreement with the epileptic phenotype and the hypersensitivity to cytotoxic agents of mice lacking TEF family members and the data presented here showing that TEF is downregulated by p53. Therefore, if p53 has been implicated in seizure neuronal damage, downregulation of TEF through a p53-mediated induction of miR-125b, could contribute to the overall apoptotic response promoted by activation of this suppressor gene. Although we must take into consideration that our data were mostly obtained using mouse fibroblasts, we may hypothesize that the miR-125b-dependent regulation of TEF contributes to the response to p53 activation in both physiological conditions (i.e., response to cellular stress stimuli) and certain degenerative diseases. The biological importance of miRs, initially demonstrated in cancer, has been extended to other diseases and the physiology of normal cells.

Although we do not know the molecular link between actin filaments and TEF, we might speculate that genetic alterations of this transcription factor could disrupt the normal cytoskeletal dynamics of neurons in a way similar to that observed in TEF (−/−) fibroblasts. Future studies will need to be done in order to establish an association between TEF or other PAR bZIP gene mutations and epilepsy or other neuronal disorders. Overall, we set the basis of a regulatory pathway orchestrated by p53 which modifies the expression levels of TEF through activation of miR-125b, and showed that this pathway has a significant influence on cytoskeletal organization and cell shape.

## Materials and Methods

### Cell cultures

Fibroblasts were obtained from tail tips (kindly provided by Dr. Ueli Schibler) of wild type and TEF knockout mice [4]. Mouse tails were minced and digested with 1 mg/ml type 1 collagenase in Dulbecco's modified Eagle's medium (DMEM) (Invitrogen, Carlsbad, CA) containing 100 units/ml penicillin and 100 µg/ml streptomycin for 1 hr at 37°C. After letting the tissue fragments settle, the supernatant was collected and one-tenth volume of fetal calf serum (FCS) (Flow Laboratories, Irvine, CA) was added to stop the collagenase action. The remaining tissue fragments were redigested for an additional 1 h and the resulting cell suspension was centrifuged and resuspended in DMEM with 10% FCS and antibiotics. Thus, each culture was derived from one tail, without any clonal selection. In all experiments, both wild type and TEF (−/−) cells were used between passages 3 and 6.

Human embryonal carcinoma cell line NTERA-2 and HEK293T cells (both from ATCC, Manassas, VA), were maintained in DMEM supplemented with 10% FCS. NB4-tsp53 leukemia cell line [34], a NB4 derivative transfected with a temperature-sensitive mutant form of p53, was grown in RPMI 1640 with 10% FCS.

To determine mitochondrial membrane potential, cells were incubated with 20 nM 3,3′-dihexyloxacarbocyanine iodide and propidium iodide for 30 min at 37°C and analyzed immediately by flow cytometry.

### Electrophoretic Mobility Shift Assay (EMSA)

Cells were lysed and nuclear fractions were resuspended in 20 mM HEPES pH 7.9, 420 mM NaCl, 1 mM EDTA, 1 mM EGTA, and 20% glycerol. Nuclear extracts (5–10 µg of total protein) were incubated with a ^32^p-labeled double stranded DNA target of TEF [6]. Samples were run on a 5% non-denaturing polyacrylamide gel in 200 mM Tris-borate, 2 mM EDTA. Gels were dried and visualised by autoradiography.

### Analyses of microRNAs

MicroRNAs miR-34a, miR-147 and miR-125b were quantified by qPCR (TaqMan MicroRNA Assay, Applied Biosystems, Foster City, CA), according to the instructions supplied by the manufacturer. The small nuclear RNA RNU6B, was used as internal control and for normalization. Amplifications were performed on a 3100 Real-Time PCR system (Applied Biosystems).

### Cell Cycle Analyses

For DNA cell cycle analysis, cells were fixed in 70% ethanol, washed in PBS and treated with 100 µg/ml ribonuclease. Then, cells were labeled with 100 µg/ml propidium iodide for 15 min and analyzed by flow cytometry (FACSCalibur, BD Biosciences) using the ModFit software.

### Analyses of mRNA expression

Total RNA was extracted using the RNeasy mini kit (Qiagen, Valencia, CA). To assess the expression of individual genes, a cDNA was generated and amplified by using primers for mouse Cdc42 ( ^5′^GCCTATTACTCCAGAGACTGC^3′^ ), and ( ^5′^GTTCATAGCAGCACACACCTG^3′^ ), RhoA ( ^5′^GTTGGTGATGGAGCTTGTGG^3′^ ), and ( ^5′^GCAGGCGGTCATAATCTTCC^3′^ ), Rac1 ( ^5′^AGGCCATCAAGTGTGTGGTG^3′^ ), and ( ^5′^CTTGTCCAGCTGTGTCCCAT^3′^ ), and β2-microglobulin ( ^5′^GGCCTGTATGCTATCCAGAAA^3′^ ) and ( ^5′^GGCGTATGTATCAGTCTCAGTGG^3′^ ). Quantitative real-time PCR was performed in a 7000 Sequence Detection System (Applied-Biosystems, Foster City, CA). The ratio of the abundance of the different transcripts to that of β2-microglobulin transcripts was calculated as 2^n^, where n is the C_T_ (threshold cycle) value of β2-microglobulin minus the C_T_ value of the target mRNA. Specificity of the desired PCR products was determined with melting curve analysis.

### Western blot analysis

Protein expression was determined by western blotting as previously described [35]. Proteins (30 to 60 µg) were separated on a 12% polyacrylamide gel, and transferred to nitrocellulose. Blots were blocked with 3% bovine serum albumin and incubated with rabbit antibodies against p53, p21 (Santa Cruz Biotechnology, Santa Cruz, CA), and TEF (Aviva Systems Biology, San Diego, CA) or mouse anti-α-Tubulin antibodies (SIGMA, St Louis, MO), and then incubated with goat anti-rabbit or anti-mouse antibodies conjugated to alkaline phosphatase (SIGMA). Bound antibody was detected by a chemiluminescence system (Applied Biosystems).

### Transfections and gene reporter assays

NTERA2 cells were stably transfected with a p53 shRNA plasmid (Mission shRNA, SIGMA) by using Lipofectamine 2000 and selected with puromycin. Transfection of NB4 cells was performed using the cell line nucleofector kit V (Lonza, Basel, Switzerland). Wild type fibroblasts were transfected with miR-34a or miR-125b precursors (Applied Biosystems) using Hyperfect. A FAM-labeled, random sequence pre-miR was used for monitoring transfection efficiency by flow cytometry.

A genomic PCR fragment of 2 kb from the promoter region of mouse TEF, was cloned into KpnI and HindIII sites of the pGL2-basic luciferase reporter vector (Promega Corp., Madison, WI). The authenticity of the construct was confirmed by sequencing. HEK293T cells were co-transfected with 1 µg of the promoter-containing pGL2 construct, 1 µg of p53 cDNA cloned in the pcDNA3 expression vector, and 20 ng of pRSV-β-gal by using Superfect. 24 h post-transfection, cell extracts were prepared and analyzed for the relative luciferase activity by a dual-light reporter gene assay system (Applied Biosystems). Results were normalized for transfection efficiency with values obtained with pRSV-β-gal.

Double-stranded oligonucleotides corresponding to the 3′ untranslated region of mouse TEF, which contain the miR-125b binding sequence ( ^5′^GAGGGTGTGTTTGTTTGCTCAGGGCGGGCAGCCT^3′^ ) or a mismatch sequence ( ^5′^GAGGGTGTGTTTGTCCGAAGCATACGGTGTGCCT^3′^ ) was cloned into XhoI and SmaI sites of the psiCHECK-1 reporter vector (Promega). HEK293T cells were transfected with these constructs (1 µg), as indicated above. pRSV-β-gal was also used to normalize transfections.

When indicated, wild type fibroblast were cotransfected with 1 µg of pCMV-DsRed (Clontech, Mountain View, CA), and 1 µg of either TEF cDNA [6] or an empty pCMV vector. After 24 h of transfection, DsRed-positive cells were sorted by flow cytometry and subsequently cotransfected with 375 ng of miR-125b and FAM-labeled miR. Transfected cells were visualized 48 h later under a fluorescence microscope.

### Immunofluorescence

Cells were assayed for the intracellular distribution of actin filaments (F-actin). Briefly, mouse fibroblasts were grown on 10×10 mm coverslips and then fixed in 3.7% formaldehyde and permeabilized with 0.5% Triton X-100. For staining actin filaments, cells were incubated with Tetramethylrhodamine isothiocyanate (TRITC)-labeled or FITC-labeled Phalloidin (SIGMA) for 20 min at room temperature. When indicated, TEF (−/−) fibroblasts were transfected with TEF cDNA, and wild type cells were transfected with miR-125b. After 48 h of transfection, fibroblasts were subjected to immunofluorescence analysis. All cell samples were mounted with VectaShield (Vector Laboratories, Burlingame, CA) and examined with a LSM-510 laser scanning microscope (Carl Zeiss Inc, Germany), equipped with an argon (488 nm) and HeNe (543 nm) ion lasers and using 40× and 63× oil (1.4 NA) objectives. Each channel was recorded independently, and pseudocolor images were generated and superimposed.

### Statistical analysis

All statistics were calculated with the SPSS statistical package (version 13.0). Categoric variables were compared using the Chi-square test. The Student t test was used to compare continuous variables, summarized as means±SD, between two groups. The significance level was set at p<0.05.
